# Statin Use Is Associated with a Decreased Risk of Mortality among Patients with COVID-19

**DOI:** 10.3390/jcm10071450

**Published:** 2021-04-01

**Authors:** Chieh-Chen Wu, An-Jen Lee, Chun-Hsien Su, Chu-Ya Huang, Md. Mohaimenul Islam, Yung-Ching Weng

**Affiliations:** 1 Department of Healthcare Information and Management, School of Health Technology, Ming Chuan University, Taipei 333, Taiwan; anjenlee@mail.mcu.edu.tw (A.-J.L.); chuya@tche.org.tw (C.-Y.H.); kimweng@mail.mcu.edu.tw (Y.-C.W.); 2 Department of Exercise and Health Promotion, College of Kinesiology and Health, Chinese Culture University, 55 Yangmingshan, Huagang Road, Shilin District, Taipei 11114, Taiwan; 3 Southeast Asian, Cross-Strait and Overseas Student Institute, Ming Chuan University, Taipei 333, Taiwan; 4 Graduate Institute of Sport Coaching Science, College of Kinesiology and Health, Chinese Culture University, Taipei 11114, Taiwan; chsu@ulive.pccu.edu.tw; 5 Taiwan College of Healthcare Executives, 9F No. 95, Sec. 3, Roosevelt Rd., Da’an District, Taipei City 106607, Taiwan; 6 Graduate Institute of Biomedical Informatics, College of Medical Science and Technology, Taipei Medical University, Taipei 11031, Taiwan; d610106004@tmu.edu.tw

**Keywords:** statin, COVID-19, SARS-CoV-2, mortality risk, diabetes

## Abstract

Background: Recent epidemiological studies remain controversial regarding the association between statin use and reducing the risk of mortality among individuals with COVID-19. Objective: The objective of this study was to clarify the association between statin use and the risk of mortality among patients with COVID-19. Methods: We conducted a systematic articles search of online databases (PubMed, EMBASE, Scopus, and Web of Science) between 1 February 2020 and 20 February 2021, with no restriction on language. The following search terms were used: “Statins” and “COVID-19 mortality or COVID19 mortality or SARS-CoV-2 related mortality”. Two authors individually examined all articles and followed the Preferred Reporting Items for Systematic Reviews and Meta-Analyses (PRISMA) guidelines for study inclusion and exclusion. The overall risk ratio (RRs) with 95% confidence interval (CI) was calculated to show the strength of the association and the heterogeneity among the studies was presented Q and *I^2^* statistic. Results: Twenty-eight studies were assessed for eligibility and 22 studies met the inclusion criteria. Statin use was associated with a significantly decreased risk of mortality among patients with COVID-19 (RR _adjusted_ = 0.64; 95% CI: 0.57–0.72, *p* < 0.001). Moreover, statin use both before and after the admission was associated with lowering the risk of mortality among the COVID-19 patients (RR _adjusted;_ _before_ = 0.69; 95% CI: 0.56–0.84, *p* < 0.001 and RR _adjusted;_ _after_ = 0.57; 95% CI: 0.54–0.60, *p* < 0.001). Conclusion: This comprehensive study showed that statin use is associated with a decreased risk of mortality among individuals with COVID-19. A randomized control trial is needed to confirm and refute the association between them.

## 1. Introduction

### 1.1. Rationale

The Coronavirus disease 2019 (COVID-19) first discovered in Wuhan, China, in December 2019, and the World Health Organization (WHO) then declared it a global pandemic on 11 March 2020 [1]. COVID-19 is caused by severe acute respiratory syndrome coronavirus 2 (SARS-CoV-2), which has created an unprecedented threat to public health worldwide [2]. As of 28 February 2021, more than 114 million confirmed cases with a mortality rate of 3% had been recorded globally (https://www.worldometers.info/coronavirus/; accessed on 28 February 2021). Older patients with multiple comorbidities are at the greatest risk of disease severity, leading to hospitalization and death. Indeed, hypertension, diabetes and cardiovascular diseases are the top three reported comorbidities and potential risk factors for COVID-19 mortality [3,4,5].

3-hydroxy-3-methyl-glutayl-CoA (HMG-CoA) reductase inhibitors, statins, are the most widely prescribed medication for the treatment of cardiovascular disease, including stroke, hyperlipidemia, and myocardial infarction [6,7,8]. Recently, epidemiological studies have highlighted the association between the statin and the reduced risk of severity and mortality among COVID-19 patients [9,10]. Statin can reduce the rate of mortality in various ways, like increasing vascular endothelial function [11], blocking miR-133a expression [12], reducing acute-phase proteins level (like C-reactive protein) [13], and anti-inflammatory effects [14].

### 1.2. Goal of This Investigation

In this present study, we conducted a comprehensive meta-analysis to assess whether statin has a beneficial effect for reducing the risk of mortality among patients with COVID-19. First, we evaluated the effect of statin before and after admission and the risk of mortality in patients with COVID-19. Subsequently, we examined the beneficial effect of statin use among intensive care unit (ICU) and non-ICU patients.

## 2. Methods

### 2.1. Search Strategy

We systematically searched online databases (PubMed, Embase, Web of Science, and Scopus) between 1 February 2020 and 20 February 2021, with no language restriction. The following search terms were used: “Statins” and “COVID-19 mortality or COVID19 mortality or SARS-CoV-2 related mortality”. We also investigated the reference lists of retrieved studies to ensure the comprehensiveness of this current study. Finally, we uploaded all studies to EndNote, a reference software for further review and selection.

### 2.2. Eligibility Criteria

Two of the authors independently checked the titles and abstracts of retrieved studies. Studies were selected if they were (a) published in English, (b) evaluated the effectiveness of statin for the mortality of patients with COVID-19, (c) published as a research article, research letter, and (d) provided the effect size with 95% CI. We excluded studies if they (a) did not provide proper definition of statin users, (b) did not provide information on how to include and exclude COVID-19 patients and statin users, and (c) did not adjust statin users with other confounding factors. Disagreements during the study selection process were resolved by discussion with a main investigation.

### 2.3. Data Extraction

The same two authors extracted all relevant information from selected studies based on prespecified guidelines. The primary outcome measures were hazard ratios (HRs), odd ratios (ORs) with 95% CI for the association between statin use and the risk of mortality among patients with COVID-19. They collected adjusted effect sizes with corresponding 95% CI for reducing confounding factors. Information regarding author, country, number of participants, rate of mortality, percentage of male, inclusion and exclusion criteria, and study design were reviewed.

### 2.4. Statistical Analysis

All analyses were conducted using Comprehensive Meta-Analysis software, version 2.2 (Biostat Inc. 14 North Dean Street, Englewood, NJ 07631, USA). The effect size was conducted using a random-effects model (REM). The risk ratios (RRs) and 95% CIs were calculated using the Peto method. A different subgroup was calculated to determine how robust the findings are. We calculated statistical heterogeneity across the various studies which was tested using the Cochran Q statistic and quantified by the I^2^ value. Funnel plot asymmetry was drawn to show publication bias.

## 3. Results

### 3.1. Article Screening

**Figure 1:**
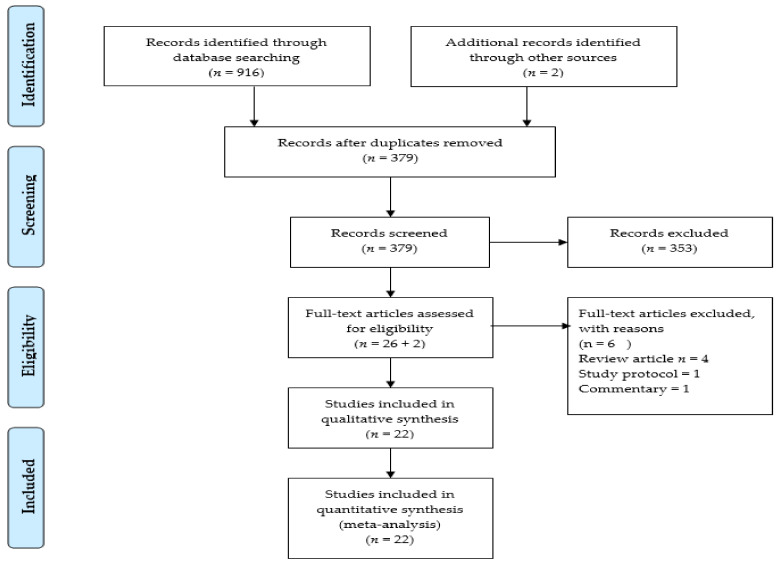
PRISMA flow diagram.

The articles search of the online databases yielded 916 articles. We excluded 537 duplicate articles and 353 articles were excluded after reviewing the titles and abstracts. We further examined 26 full-text articles and searched the references of relevant articles, retrieving 2 additional publications. Finally, 22 studies met all inclusion criteria [1,9,10,15,16,17,18,19,20,21,22,23,24,25,26,27,28,29,30,31,32,33]. The flow diagram of the systematic articles search is presented in Figure 1.

### 3.2. Study Characteristics

Table 1 shows the characteristics of the included studies. All the studies retrospectively collected data. Nine studies were published from North America, 8 studies from Europe and 5 studies from Asia. Seventeen studies reported that statin was used in non-ICU patients and 5 studies reported the use of statin in ICU patients. However, 14 studies demonstrated that the use of statin before being hospitalized was associated with decreased risk of mortality among patients with COVID-19.

### 3.3. Statin Use and Risk of Mortality

**Figure 2:**
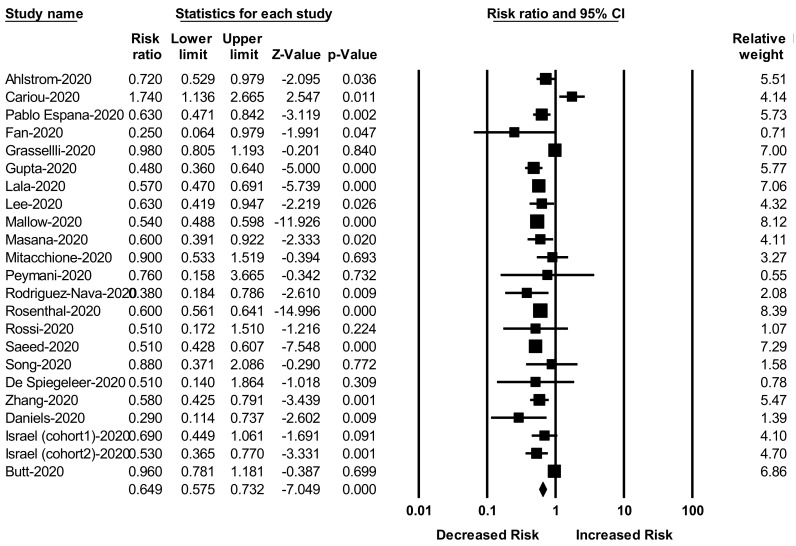
Forest plot for the studies on the association between statin use and risk of COVID-19 mortality.

Our meta-analysis consists of 22 studies with COVID-19 participants. Statin use was associated with a significantly decreased risk of mortality among patients with COVID-19 (RR _adjusted_ = 0.64; 95% CI: 0.57–0.73, *p* < 0.001) (Figure 2). The heterogeneity among the studies was moderate and significant (*I*^2^ = 74.96, Q = 87.86, and τ^2^ = 0.04).

### 3.4. Secondary Analysis

#### Statin Use before and after Admission and the Risk of Mortality

**Figure 3:**
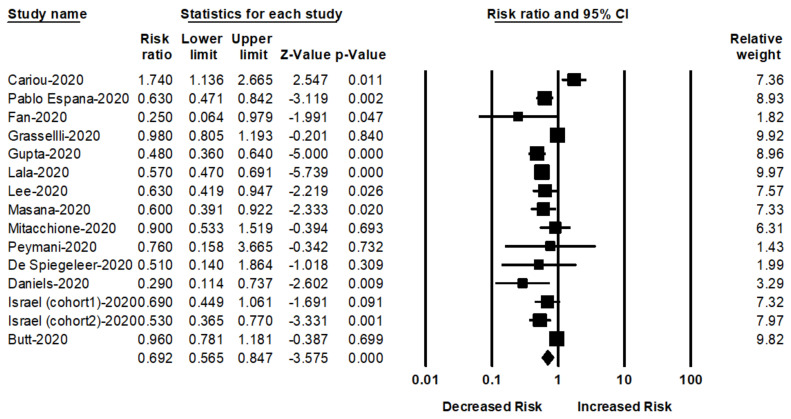
Forest plot for the studies on the association between statin use before admission and risk of COVID-19 mortality.

We pooled 14 studies in order to evaluate the impact of statin use before admission and the risk of mortality among patients with COVID-19. The pooled risk ratio of mortality was 31% lower in statin users than that of without statin users (RR _adjusted_ = 0.69; 95% CI: 0.56–0.84; *I*^2^ = 75.89%) (Figure 3).

**Figure 4:**
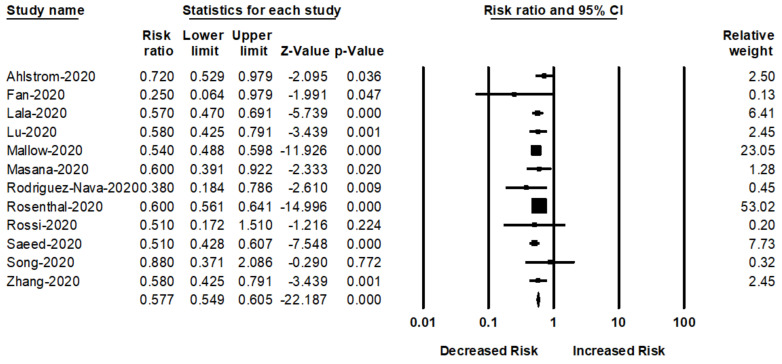
Forest plot for the studies on the association between statin use after admission and risk of COVID-19 mortality.

We pooled 11 studies in order to evaluate the impact of statin use after admission and the risk of mortality among patients with COVID-19. The pooled risk ratio of mortality was 43% lower in statin users than that of without statin users (RR _adjusted_ = 0.57; 95% CI: 0.54–0.60; *p* < 0.001) (Figure 4). There was no heterogeneity among the studies (*I*^2^ = 5.63, Q = 10.59, and τ^2^ = 0.001).

### 3.5. Subgroup Analysis

Subgroup analyses are presented in Table 2. Seventeen studies reported the risk of mortality among non-ICU patients with statin and identified the pooled RR of 0.63 (95% CI: 0.56–0.70, *p* < 0.001). Five studies also reported the risk of mortality among ICU patients with statin and identified the pooled RR of 0.66 (95% CI: 0.48–0.92, *p* = 0.01). Nine studies from Europe evaluated the beneficial effect of statin among individuals with COVID-19. The pooled risk ratio of mortality was 0.84 (95% CI: 0.68–1.04, *p* = 0.12). However, the pooled risk ratio of mortality among the patients from North America and Asia was 0.55 (95% CI: 0.50–0.60, *p* < 0.001), and 0.58 (95% CI: 0.50–0.68, *p* < 0.001), respectively.

### 3.6. Publication Bia

**Figure 5:**
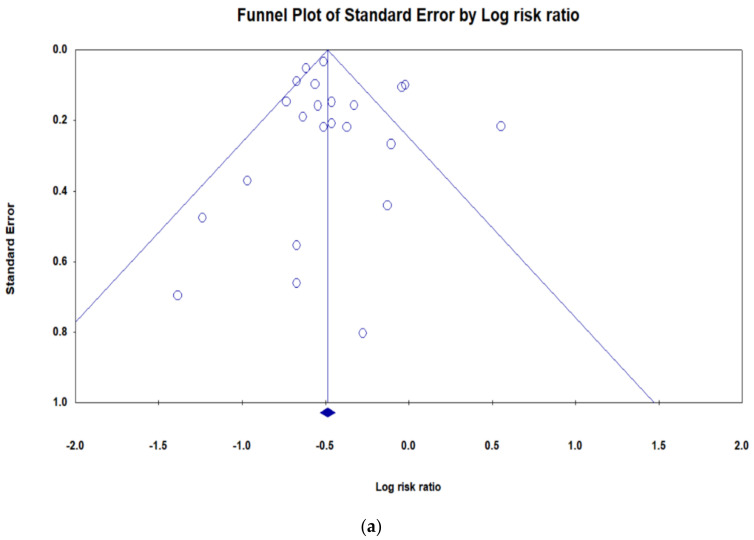

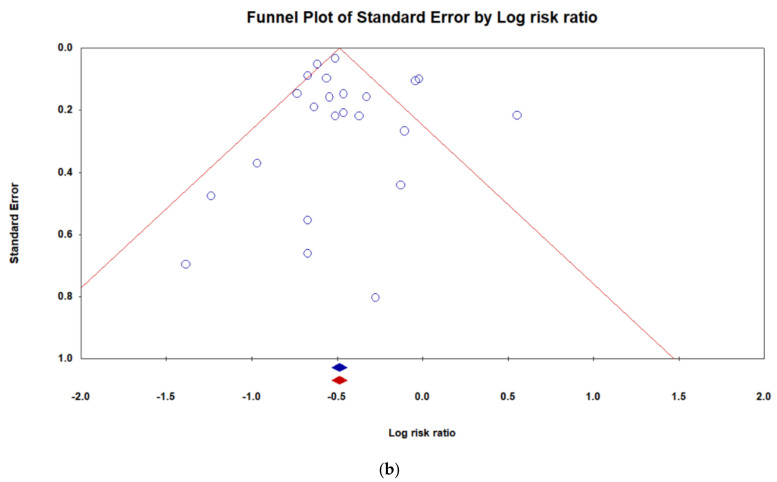
Funnel plot (**a**); Funnel plot by trim and fill method (**b**).

Figure 5a shows the funnel plot clustering of studies. Egger’s test confirmed asymmetry of the funnel plot (0.61; 95% CI: −0.88, 1.69; *p* = 0.51) and shows no publication bias. Figure 5b shows the funnel plot with missing studies imputed by the trim-and-fill method. There was no missing study to be filled in the plot, and the overall log risk ratio became 0.61 95% CI (0.58–0.64).

## 4. Discussion

### 4.1. Main Findings

The comprehensive meta-analysis of 22 studies shows a 36% lower risk of mortality among COVID-19 patients with statins. Use of statin both before and after the admission was associated with a reduced risk of mortality (0.69 and 0.57). Statin usage in North American and Asian patients with COVID-19 was associated with lower mortality than European. A previous study also showed the favorable effect of statin in North American and European patients with COVID-19 [34]. Indeed, treating COVID-19 patients with statins could help to reduce in-hospital mortality.

### 4.2. Comparison with Other Studies

Our study findings are similar to three previously published studies. Kow et al. [35] included only 5 studies to examine the beneficial effect of statin in COVID-19 patients. The pooled analysis showed a significantly decreased risk of severity/mortality among COVID-19 patients with statins (HR = 0.70; 95% CI: 0.53–0.94) compared to non-statin users. Permana et al. [36] conducted a meta-analysis of 13 studies with 52,122 patients. Their study examined the effect of statin use in pre-and in-hospital admission and the risk of mortality among COVID-19 patients. In-hospital use of statin was significantly associated with a lower risk of mortality (RR: 0.54; 95% CI: 0.50–0.58, *p* < 0.001); although, the use of statin before hospital admission was not associated with risk of mortality (RR: 1.18; 95% CI 0.79–1.77, *p* = 0.41). Finally, Chow et al. [37] also evaluated the protective effect of statin on patients’ mortality with COVID-19 by analyzing 13 studies. Statin use before and after hospitalization showed a protective effect on a lower risk of mortality (HR: 0.80, 95% CI: 0.50–1.28 vs. 0.53; 95% CI: 0.46–0.57). This updated meta-analysis included a total of 22 articles with 217,441 participants. Furthermore, this study is more informative; it shows secondary and subgroup analyses.

### 4.3. Possible Biological Mechanisms

Several possible biological plausibilities exist on how statin reduces the risk of mortality among patients with COVID-19. First, inflammation induces mortality in patients with COVID-19; however, statin has anti-inflammatory and immunomodulatory effects. Second, statin can lessen the activity of SARS-CoV-2 infection by inhibiting its’ main protease [38]. Third, it has been reported that the level of angiotensin II was higher in patients with COVID-19, and it reflects the association with viral load and severity of illness [39]. SARS-CoV-2 viruses utilize ACE2 for cell entry, and unbalance the ACE2 expression by binding the viral spike protein to the ACE2 receptor [40]. ACE2 downregulation then produces excessive angiotensin, which is reported to be associated with severe respiratory failure [40]. Previous studies already highlighted the role of statin to upregulate ACE2 [41,42]. However, more biological studies are needed to confirm the association between them.

### 4.4. Strengths and Limitations

Our study has several strengths. First, this is the most up-to-date meta-analysis that showed the beneficial effect of statin for reducing the risk of mortality among patients with COVID-19. Second, this study showed secondary and subgroup analyses that were missing in the previously published literatures. Third, our findings show minimum heterogeneity, and bias among the studies was absent. Our study has several limitations that also need to be addressed. First, our analysis does not explore the duration of statin and the risk of mortality among patients with COVID-19. Second, it does not show statin dose, the severity of disease and the risk of mortality due to a lack of data.

## 5. Conclusions

Our study findings support the notion that statin use is associated with a reduced risk of mortality in patients with COVID-19. The results of this study have also highlighted the need for further randomized control trials exploring the utility of statin to reduce the mortality of patients with COVID-19.

## Figures and Tables

**Table 1:** The characteristics of included studies.

Author	Country	Study Design	Study Period	No. of Patients	Age (Mean/Median)	Sex, Male	% Statin Users	Statin Use after (A) or Before (B) Admission	Patient Criteria	Inclusion Criteria	Results	Outcome
Ahlström	Sweden	Retrospective cohort	27 May 2020	9905	61	74	26.1	A	ICU	ICD-10	0.72 (0.53–0.98)	In-hospital
Butt	Denmark	Retrospective cohort	22 February–17 May	4842	54	47.1	17.4	B	Non-ICU	ICD-10	0.96 (0.78–1.18)	All-cause mortality
Cariou	France	Retrospective cohort	10 March–10 April	2449	70.9	64.02	48.7	B	Non-ICU	Radiology	1.74 [1.13–2.65]	In-hospital
Pablo España	Spain	Retrospective cohort	February–22 May	11,261	84.15	30.33	N/A	B	ICU	ICD-10	0.63 (0.47–0.84)	In-hospital
Fan	China	Retrospective cohort	12 March–14 April	412	64	43.7	11.64	AB	ICU	Hospital record	0.25 (0.06–0.92)	In-hospital
Grasselli	Italy	Retrospective cohort	20 February–22 April	5914	63	79.9	17.7	B	ICU	Hospital record	0.98 (0.81–1.20)	In-hospital
Gupta	USA	Retrospective cohort	1 February–11 June	2626	69	56.5	36.2	B	Non-ICU	ICD-10	0.48 (0.36–0.64)	30 day mortality
Lala	USA	Retrospective cohort	27 February–12 April	2736	66.4	59.6	36	AB	Non-ICU	Hospital record	0.57 (0.47–0.69)	In-hospital
Lee	Korea	Retrospective cohort	19 January–16 April	10,448	65.53	33.6	5.10	B	Non-ICU	ICD-10	0.637 [95% CI, 0.425–0.953]	60 day mortality
Mallow	USA	Retrospective cohort	15 March–30 April	21,676	64.9	52.8	24.5	A	Non-ICU	ICD-10	0.54 (0.49–0.60)	In-hospital
Marsana	Spain	Retrospective cohort	N/A	2157	67	57.2	24.5	AB	Non-ICU	Hospital record	0.60 (0.39–0.92)	In-hospital
Mitacchione	Italy	Retrospective cohort	23 February–31 March	842	64	62	21.25	B	Non-ICU	Hospital record	0.90 (0.53–1.51	In-hospital
Peymani	Iran	Retrospective cohort	1 March–30 May	459	65	62.7	50	B	Non-ICU	Hospital record	0.76 (0.16–3.72)	In-hospital
Rodriguez-Nava	USA	Retrospective cohort	March–May 2020	87	68	64.4	N/A	A	ICU	Hospital record	0.38 (0.18–0.77)	In-hospital
Rosenthal	USA	Retrospective cohort	1 April–1 May	64,781	56.1	49.3	18.9	A	Non-ICU	ICD-10	0.60 (0.56–0.65)	In-hospital
Rossi	Italy	Retrospective cohort	29 February–15 June	71	71	57.1	59.1	A	Non-ICU	Hospital record	0.51 (0.17–1.49)	In-hospital
Saeed	USA	Retrospective cohort	1 March–2 May	4252	65	53	46.7	A	Non-ICU	Hospital record	0.51 (0.43–0.61)	In-hospital
Song	USA	Retrospective cohort	3 March–10 April	249	62	57	49.4	A	Non-ICU	Hospital record	0.88 (0.37–2.08)	In-hospital
De Spiegeleer	Belgium	Retrospective cohort	1 March–16 April	154	85	33	20.1	B	Non-ICU	Hospital record	0.51 (0.14–1.35)	In-hospital
Daniels	USA	Retrospective cohort	10 February–17 June	170	59	58	48.7	B	Non-ICU	Hospital record	0.29 (0.11–0.73)	In hospital
Zhang	China	Retrospective cohort	N/A	13,981	66	49.4	8.71	A	Non-ICU	Hospital record	0.58 (0.43–0.80)	30 day mortality
Israel	Israel	Retrospective cohort	25 September–10 October	57,969	N/A	48.9	8.7	B	Non-ICU	Hospital record	0.69 (0.44–1.06) & 0.53 (0.36–0.77)	In-hospital

**Table 2:** Summary of subgroup analyses.

Subgroup	No. of Study	Effect Size	95% CI	*p*-Value	*I* ^2^	Q-Value	τ^2^
**Hospital stay**							
**Non-ICU**	17	0.63	0.56–0.70	<0.001	70.20	60.40	0.03
**ICU**	5	0.66	0.48–0.92	0.01	71.28	13.92	0.07
**Regions**							
**Europe**	9	0.84	0.68–1.04	0.12	65.37	23.10	0.05
**North America**	8	0.55	0.50–0.60	<0.001	33.15	10.47	0.004
**Asia**	5	0.58	0.50–0.68	<0.001	0	2.56	0
